# IgG4 induces tolerogenic M2-like macrophages and correlates with disease progression in colon cancer

**DOI:** 10.1080/2162402X.2021.1880687

**Published:** 2021-02-08

**Authors:** Galateja Jordakieva, Rodolfo Bianchini, Daniel Reichhold, Jakob Piehslinger, Alina Groschopf, Sebastian A. Jensen, Ettore Mearini, Giuseppe Nocentini, Richard Crevenna, Gerhard J. Zlabinger, Sophia N. Karagiannis, Alexander Klaus, Erika Jensen-Jarolim

**Affiliations:** a Department of Physical Medicine, Rehabilitation and Occupational Medicine, Vienna, Austria; b The Interuniversity Messerli Research Institute of the University of Veterinary Medicine, the Medical University of Vienna and the University of Vienna, Unit of Comparative Medicine, Vienna, Austria; c Institute Pathophysiology and Allergy Research, Center for Pathophysiology, Infectiology and Immunology, Division of Comparative Immunology and Oncology, Medical University of Vienna, Vienna, Austria;; d Department of General Surgery, Barmherzige Schwestern Krankenhaus Wien, Vienna, Austria; e FH Campus Wien, Department of Health Science, Section of Biomedical Analytics, University of Applied Sciences, Vienna, Austria; f Department of General Surgery, Medical University of Vienna, Vienna, Austria;; g Department of Surgical and Biomedical Sciences, Urology Clinic of Perugia, University of Perugia, Perugia, Italy;; h Section of Pharmacology, Department of Medicine, University of Perugia, Perugia, Italy; i Division of Clinical and Experimental Immunology, Institute of Immunology, Center for Pathophysiology, Infectiology and Immunology, Medical University of Vienna, Vienna, Austria;; j St. John’s Institute of Dermatology, School of Basic & Medical Biosciences, King’s College London, London, UK; k Breast Cancer Now Research Unit, School of Cancer & Pharmaceutical Sciences, King’s College London, Guy’s Cancer Centre, London, UK

**Keywords:** IgG4, IgE, tumor-associated macrophages, M2b, allergooncology

## Abstract

IgG4 subclass antibodies are expressed in alternative Th2 environments featuring high IL-10 expression, including several solid tumors such as melanoma. To induce tolerance, allergen immunotherapy mediates antibody class switching from pro-inflammatory IgE to anti-inflammatory IgG4. We previously reported that IgG4 drives allergic M2 macrophages toward tolerogenic states. Here we assessed the roles of IgG4 and macrophage activation in colorectal cancer (CRC).

In this observer-blinded, case-control study, we analyzed total circulating serum IgE, IgG1 and IgG4 levels in CRC (n = 38) patients with (n = 13, TxNxM1) or without (n = 25, TxNxM0) metastasis, and in healthy donors (n = 21). Primary cultures of circulating monocyte-derived macrophages from healthy controls and CRC patients were further evaluated in their responses to stimulation with IgG1 or IgG4.

We found higher absolute serum levels of IgG4 in patients with CRC. IgG4 enabled polarization of macrophages derived from CRC patients and healthy controls into alternatively-activated tolerogenic M2b phenotypes. IgG4-stimulated M2 macrophages were characterized by lower surface CD206, CD163, CD14, and CD11b expression and higher CCL-1, IL-10, and IL-6 production. IgG4 was less potent that IgG1 in triggering antibody-dependent cell-mediated phagocytosis (ADCP) of cancer cells. Further, higher z-normalized IgG4/-IgE sera level ratios correlated with the presence of metastasis (*p* = .0247 and *p* = .0009, respectively) in CRC patients.

High IgG4 in CRC synergizes with macrophages in shaping an immunosuppressive microenvironment and impairs anti-cancer effector cell functions. The shift of serum IgG4/IgE ratios toward enhanced tolerance induction in metastatic disease indicates a role for high IgG4 in disease progression and poor prognostic outcome.

## Introduction

Immunoglobulin E (IgE) induces a strong activation of Fc epsilon receptors (FŒRs) and can activate cytotoxic innate effector cells, such as eosinophils, monocytes, macrophages, and mast cells. These functions are widely established in allergic inflammation. In cancer, besides the debated roles of mast cells, ^1^ tumor-associated tissue eosinophilia (TATE) and macrophage (TAM) infiltration are considered characteristics of on-site inflammation .^2–6^ These infiltrates could however contribute to anti-tumor responses via antibody-dependent cell-mediated cytotoxicity (ADCC) and phagocytosis (ADCP) when stimulated by IgE. Furthermore, accumulation of IgE directed to cancer tissues can contribute to anti-tumor inflammation stimulated by highly-expressed cancer antigens form tumor-associated molecular patterns (TAMPs), facilitating IgE cross-linking and thereby triggering release of cytotoxic macrophage and mast cell-derived mediators, such as TNF-α. Both effector and immune-stimulating functions can induce anti-tumor effector cell activation .^7^

A significant portion of tumor-infiltrating lymphocytes, however, have been identified as regulatory T cells (T_regs_) or regulatory B cells (B_regs_), which are reported to actively suppress immune responses. They create an immunomodulatory microenvironment by production of immunosuppressive mediators, such as IL-10. IL-10 on the one hand indirectly downregulates IgE production, ^8^ while on the other hand it accelerates IL-4 mediated isotype switch toward IgG4 production by B cells .^9^ IL-10 is therefore critical in tipping the balance toward a low IgE/IgG4 ratio. This is well known in the context of IgE-mediated allergy, where allergen immunotherapy typically shifts the response toward IgG4 production, a process known to be associated with immune tolerance toward the allergen. In contrast to IgE, IgG4 induces the weakest activation of FcγRs on effector cells out of all immunoglobulins, and it is the only IgG subclass/isotype which does not activate the complement system .^10^ Previous studies in melanoma^11^ and preliminary findings in CRC indicate that IgG4 is expressed in cancer tissues .^12,13^ Irregular serum IgG4 levels and concurrent diagnosis of IgG4-related diseases have been reported in several cancer types .^14–18^ Abundant IgG4+ plasma cell infiltrates were described in pancreatic cancer tissues^19^ and IgG4 was found to be the dominant IgG subclass in both serum and tumor tissue of patients with thyroid carcinoma, ^20,21^ where IgG4+ plasma cells were associated with unfavorable prognostic aspects, such as multi-focality. Further, in gastric cancer, higher numbers of IgG4-positive cells in tumor tissues were associated with disease progression and poor prognostic outcomes .^22^

In cancer tissues, approximately 50% of infiltrating immune cells have been identified as macrophages, often polarized to the alternatively-activated “M2” phenotype .^4^ We have previously shown that the (“alternatively-activated”) subtype M2a, which is prominent in IgE-mediated allergy, is repolarized to an immune-regulatory (“type II activated”) phenotype M2b in the presence of IgG4 .^23^ It is known that M2 macrophages may create a pro-tumorigenic microenvironment^2,24–26^ which correlates with angiogenesis, ^25,27,28^ survival and proliferation of tumor cells .^3,27^ However, the macrophage subtype responsible for these modulatory signals has not been defined consistently. In line with the AllergoOncology concept [27, 28], we transferred and adapted our experiments with alternatively differentiated macrophages from the allergy field into oncology. Here we evaluated whether IgG4, previously reported to be overexpressed in melanoma and other cancers with poor outcomes, can stimulate M2a and M2b macrophages to adapt immunosuppressive states and to enhance production of IL-10 and other immunomodulatory mediators. We asked whether IgG4 can support a pro-tumorigenic microenvironment by driving TAMs into immune-modulating phenotypes and by disruption of antibody-dependent cell-mediated cytotoxicity (ADCC) and phagocytosis (ADCP). We furthermore aimed to assess whether these findings could be confirmed and correlated with disease progression in colorectal cancer (CRC) and, potentially, in other cancer cohorts.

## Materials and methods

### Study population

The study was conducted between 2016 and 2018 at the Department of General Surgery, St. Vincent Hospital Vienna (“Barmherzige Schwestern Wien”), Austria, after approval by the hospital’s Ethics Committee Board (Nr. 201604_EK01). The participants were either in hospital for an elective colonoscopy (controls) or for staging and/or surgery in recently diagnosed colorectal cancer (CRC patients) with (=TxNxM1) and without (=TxNxM0) systemic metastasis at initial diagnosis. Clinical characteristics (e.g. TNM stage, drug and allergy anamnesis), laboratory parameters, and follow-up data (e.g. mortality) were obtained from surgical and pathological records. The blood samples were rapidly sent to our research lab for analysis by a blinded study member (RB).

In total, 66 patients (N = 66) were recruited at the in-patient ward of the Department of General Surgery, St. Vincent Hospital Vienna, Austria. Blood samples were obtained after gaining written informed consent and rapidly analyzed in a blinded manner as described above. Six (n = 6) patients were excluded due to flaws in blood sampling or pre-analysis. One patient (n = 1), classified as M0 upon inclusion and diagnosed with peritoneal cancerosis during surgery, was excluded from further analysis, as systemic metastasis had not been established at initial diagnosis and mode of metastasis, i.e. local versus hematogenic, was unclear. Nine (n = 9) patients were excluded from the analyses because of IgE-mediated allergy and five (n = 5) patients had insufficient viable PBMCs in serum for consequent analyses. Thus, forty-five (n = 45) patients (62.2% male; age 67.4 ± 11.1 years) were eligible for analyses:

- Non-CRC patients with normal colonoscopy (=controls) (n = 15);
- CRC patients (n = 30):

1. without systemic metastasis at initial diagnosis (=TxNxM0) (n = 20);
2. with systemic metastasis at initial diagnosis (=TxNxM1) (n = 10)

### Inclusion criteria:

• age between 18 and 85 years

• recent initial CRC diagnosis, without any preceding or neo-adjuvant therapy (CRC patients)

• normal colonoscopy during the last month (controls)

• adequate German language skills

• signed declaration of consent

### Exclusion criteria:

• innate or acquired immune deficiencies

• ongoing potentially immune-modulating therapies

• active infectious or inflammatory (bowel) disease

• any other kind of malignancy in patient history

• previously diagnosed IgG4-associated disease and/or known serum IgG4 elevation.

### Immunohistochemistry (IHC) and Immunofluorescence (IF) staining

For immunostaining protocols, were prepared paraffin blocks from 13 CRC patients using the resection tissues. The 13 CRC patients derived specimens do not overlap with the recruited study population investigated in any other protocol. Serial 4-μm sections were on a microtome (Leica) cut from CRC tumor samples previously fixed in buffered 3.9% formalin and paraffin-embedded. Sections were deparaffinated in Xylene for 20 minutes and rehydrated by serial incubations in alcohol. Heat-induced antigen retrieval was performed in a pressure steam cooker with Tris-EDTA buffer, pH 9 during 15 min. Permeabilization was done with PBS 0.2% Tween20 for 15 min, and blocking with 5% FCS in PBS, for 30 min at RT. For immunohistochemistry method, DAKO EnVision+, Peroxidase system (DAKO, Glostrup, Denmark) anti-mouse was used. Sections were counterstained with hematoxylin for nuclear visualization. Alternatively, a fluorescent triple staining was performed using: 1) mouse anti-human IgG4 clone HP6025 1:500 (Genway Biotech); 2) rabbit anti-human CD38 clone EPR4106 dilution 1:100 (Abcam); 3) goat anti-human CD68 clone C-18: sc-7082 dilution 1:100 (Santa Cruz Biotechnology). The secondary antibody used were: 1) donkey anti-mouse IgG AF568 (Abcam); 2) donkey anti-rabbit IgG AF488 (Abcam); 3) donkey anti-goat IgG AF647 (Abcam). Samples were counterstained with DAPI (1:1000 dilution) and mounted in Moviol (Sigma-Aldrich). TissueFAXS (TissueGnostics, Vienna, Austria), a fully automated multi-channel immunofluorescence tissue analysis system was used for the acquisition of diseased specimen. For acquisition, the 20x/0.5 or the 40x/1.3-oil objectives were used (EC Plan_NeoFluar, Zeiss) .^29^ Filter sets were from Chroma TechnologyCorp (DAPI 350/460 nm; FITC/Cy2 470/525 nm; mCherry/TxRed 560/630 nm; Cy5 620/700 nm). Images were processed using Java image processing program Fiji software. IgG4 positive expression on CRC tumor samples were evaluated by HistoQuest software (TissueGnostics, Vienna, Austria) .^30^

### Serum levels measurement of total IgG1, IgG4 and IgE

Human peripheral blood was obtained by venipuncture using coated with lithium heparin (Greiner Bio-One, Austria) vacuum tubes CAT serum separator clot activator (Greiner Bio-One, Austria) or coated with lithium heparin (Greiner Bio-One, Austria) and then transferred in 2 ml tubes. In both the procedures the samples were allowed to clot for at least 30 min at room temperature in an upright position and were then centrifuged at 1800xg for 10 min. Serum was recovered and stored in fresh 1.5 ml tubes at −20°C. Serum levels of total of IgG1, IgG4, and IgE were determined on a BNII nephelometer (Siemens Healthcare Diagnostic Products, Vienna, Austria). The measured concentrations of total sera IgG1, IgG4, and IgE were normalized using a z-normalization formula for each class separately.

$$z=\frac{x-\overset{ˉ}{x}}{\sigma }$$

$\overset{ˉ}{x}$= mean of total sera concentration for the specific class; σ = standard deviation of total sera concentration for the specific class.

We obtained three values: z-normalized IgG1 (zIgG1) values, z-normalized IgG4 (zIgG4) values, and z-normalized IgE (zIgE) values.

Then, we hypothesized that the zIgG4 values could correlate positively with the metastatic stage of CRC and zIgE values could correlate negatively with the metastatic stage of CRC. To demonstrated that we created the zIgG4/-zIgE ratio that could include both the characteristics. As controls, zIgG1/-zIgE ratios were used.

### Purification and treatment of monocytes from peripheral blood mononuclear cells (PBMCs)

Peripheral blood mononuclear cells (PBMCs) were obtained from lithium heparin samples and monocyte cells were purified from the other cells by plastic adhesion method as previously described .^23^ Briefly, PBMCs were isolated by Ficoll-Paque (GE Healthcare, Solingen, Germany) (density 1.077 g/ml) gradient centrifugation (400 × g, 30 min, 20°C, in a swinging-bucket rotor without brake). The white layer of PBMCs was recovered and washed 3 times in RPMI 1640. After the last wash the cells were resuspended and seeded in 6 well plates (Falcon, Corning, NY) at a concentration of 2 × 10^6^ cells/ml, 3.5 ml/well. After 2 hours, non-adherent cells were washed away 2 times and adherent cells (monocytes) were maintained in RPMI 1640 with 10% heat-inactivated FBS, and 1% of P/S (cRPMI), supplemented with 20 ng/ml rh-M-CSF for 7–9 days. Half of the medium was refreshed every 2–3 days. Purity of monocyte cells was assessed after 3 days of culture by multicolor staining of CD3, CD11b, and CD86, using the SYTOX™ Green Ready Flow™ Reagent (Thermo Fisher Scientific, Waltham, MA, USA) to exclude dead cells^23^(data not shown).

### Macrophage polarization and stimulation with IgG1 or IgG4 immune complex

Adherent monocytes acquired the mature macrophage characteristics after 7–9 days of *in vitro* culture with 20 ng/ml rh-M-CSF. Macrophages were then detached using ice-cold PBS^−Ca-Mg^ supplemented with 2.5 mM EDTA (PBS/EDTA) pH 8.0 and allowed to recover in cRPMI. To mimic the creation of IgG1 or IgG4-related immune complexes (ICs) we used plate-bound human myeloma IgG1 (mIgG1) or IgG4 (mIgG4) (Athens Research and Technology, Athens, GA) as described in previous study .^23^ Briefly, a 96 well plate (Falcon, Corning, NY) was coated with mIgG1 or mIgG4 antibodies, 5 µg/well in HBSS (50 µg/ml), at 37°C, 5% CO2, 95% humidity for 1 h, and then washed twice with 200 µl of cRPMI. Then, macrophages were seeded in the wells coated or not with ICs at a cell concentration of 1.5 × 10^5^ cells/ml. To polarize the cells in M2a macrophages were used cRPMI supplemented with 20 ng/ml rh-M-CSF, 20 ng/ml rh-IL-4, and 20 ng/ml rh-IL-13.

### Staining and flow cytometric analysis

After 72 h, M2a cells incubated with or without IgG1- or IgG4-ICs were detached using ice-cold PBS/EDTA and washed twice with cell staining buffer (cat.420201 BioLegend, San Diego, CA), for surface marker phenotypization. Then, cells were incubated with a multicolor staining mix of monoclonal antibodies against CD14, CD86, CD11b, CD163, and CD206 or their isotype controls (BioLegend, San Diego, CA) diluted 1:100 in staining buffer for 30 min at 4°C followed by 2x washing with staining buffer. Samples were acquired by FACS Canto II (Becton Dickinson, Franklin Lakes, NJ) and analyzed with the FlowJo^TM^ Software version 10.3 (FlowJo, LLC, Ashland, OR, USA). Several FlowJo plugins were used to improve the Flow cytometer data analyses. First, the *FlowAI* plugin was used to identify anomalous events and clean the data by removing them. Second, the *DownSample* plugin was used to reduce the number of events necessary for the representation of all the parameters datasets in a two-dimensional space to 9.000 events. Finally, a machine learning algorithm uniform manifold approximation and projection (UMAP) was used for dimensionality reduction by using the *UMAP* plugin. For the characterization of each parameter the geometric mean fluorescence intensity (MFI) values that were calculated for each fluorochrome were used. The z‐normalization of MFI for each staining antibody and each donor was performed for M2a, M2a + IgG1, and M2a + IgG4.^23^ (Supporting information for z-norm calculation).

### ELISA

Supernatants from M2a cells either incubated on IgG1- or IgG4-IC-coated plates or not (see above) were collected after 72 h and IL-10, IL-6, and TNFα were analyzed by ELISA (Thermo Fisher Scientific, Waltham, MA), IL-12p70 by ELISA (BioLegend, San Diego, CA), and CCL1 by ELISA (R&D Systems, Minneapolis, MN, USA), following the supplier’s instructions. The z‐normalization of the concentration for each cytokine/chemokine and each donor was performed for M2a, M2a + IgG1, and M2a + IgG4.

### Cells used for the antibody-dependent cell-mediated cytotoxicity/phagocytosis (ADCC/ADCP) assay

The human epidermal epithelial carcinoma cell line A431 (ATCC CRL-1555), and the human colon adenocarcinoma cell line CaCo2/TC7 (ATCC HTB-37) were maintained in high glucose DMEM (4500 g/l glucose). The medium for CaCo2/TC7 was supplemented with 20% heat-inactivated FBS, 2 mM L-glutamine, 1 mM NEAA and 1 mM of P/S (cDMEM_CaCo2). The medium for A431 is similar to cDMEM_CaCo2 with the exception of 10% heat-inactivated FBS, and 4 mM L-glutamine (cDMEM_A431); the human colon adenocarcinoma cell line HT-29 (ATCC HTB-38), and the human myelomonocytic cell line U937 (ATCC CRL-1593.2) were maintained in RPMI 1640, supplemented with 10% heat-inactivated FBS, 1 mM of P/S, and 1 mM NEAA (cRPMI). All the cell lines were incubated at 37°C in 5% CO_2_ atmosphere .^31^

### Target cell preparation for ADCC and ADCP

**Figure 1.**
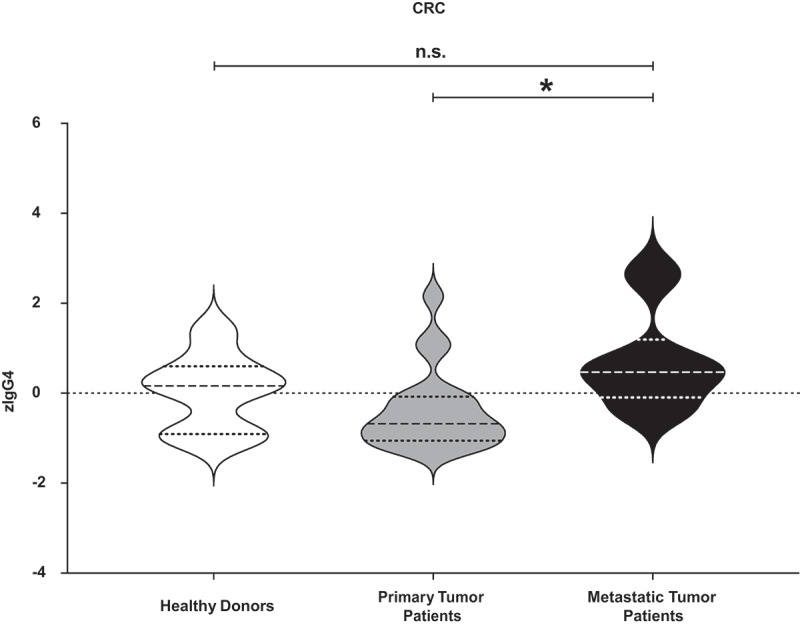
**Measurement of total IgG4 sera levels in all the different cluster of donors**. Total IgG4 levels of healthy donors, primary cancer patients and metastatic patients were measured. After z-normalization significantly higher total IgG4 sera levels (zIgG4) were found in metastatic compared to primary tumor patients (*p* = .01109). One-way ANOVA with Tukey post-hoc test was performed. n.s. *P* > .05, * *P* < .05

The human epidermoid carcinoma cell line A431, the human colon adenocarcinoma cell line HT-29, and the human colon adenocarcinoma cell line CaCo2/TC7 express different levels of EGFR (Repository Figure 1a). For CFSE staining, human cancer cell lines were detached with Accutase solution (Sigma-Aldrich, St. Louis, MO), following the supplier’s instructions. The resulting cells were washed in HBSS and incubated (3x10^6^ cell/ml) with 1 µl of 5 mM CFSE each ml for 20 min at 37°C and kept protected from light (Repository Figure 1b). After the staining period, the cells were washed with cRPMI by adding 5 times the original staining volume. Then, the cells were counted and seeded in 24-well plate at a cell concentration of 3.5 × 10^5^ cells/ml and left adhere O.N.

### Effector cell preparation for ADCC and ADCP

U937 monocytic cells were stained with 1:100 anti-FcαRI (CD89) APC (BioLegend, San Diego, CA) at 4°C after the killing assay. The anti-FcαRI (CD89) APC used for the staining of monocytic cells was labeling only the effector cells and not the tumor target cells (Repository Figure 1c).

### Cell incubation and labeling for the cytotoxicity/phagocytosis assay

The CFSE-labeled tumor target cell lines in the 24 well plate, were incubated with 20 µg/ml of cetuximab derived anti-hEGFR-IgG1 or -IgG4 subclasses/isotypes (InvivoGen, Toulouse, France), (or myeloma mIgG1 and mIgG4 as control) and with U937 monocytic cells at a cell concentration of 3.5 × 10^5^ cells/ml for 2 hours at 37°C and 5% CO^2^. The tumor cells and the monocyte/macrophage cells were seeded at a 1:1 ratio. After the incubation time, all the cells were detached with ice-cold PBS/EDTA and then washed with HBSS. The resuspended cells were incubated for 10–15 minutes at RT, protected from light with Zombie Violet™ (BioLegend, San Diego, CA) to assess live vs. dead status of the cells. Without washing, the cells were stained with anti-FcαRI (CD89) APC (BioLegend, San Diego, CA) at RT for 15 minutes. After that 0.5 ml of cell staining buffer (BioLegend, San Diego, CA) was used to wash the cells and then the cell pellet was resuspended in 200 µl of cell staining buffer and then the samples were acquired using an FACS CANTO II flow cytometer (Becton Dickinson, Franklin Lakes, NJ).^31^

### Flow cytometric analysis

**Figure 2.**
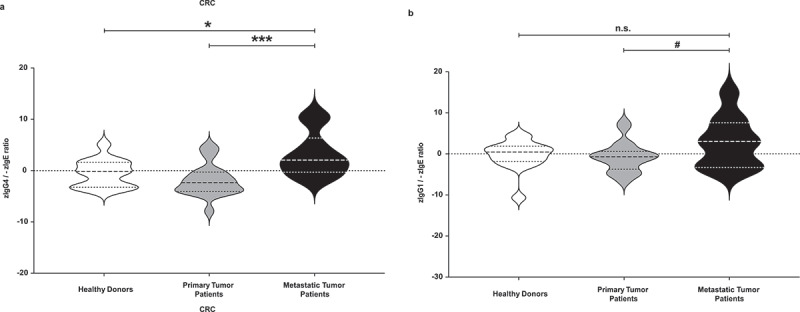
**Evaluation of total zIgG4/-zIgE ratio and of total zIgG1/-zIgE ratio**. Total IgG4, IgG1 and IgE levels of healthy donors, primary cancer patients and metastatic patients were measured. After z-normalization of all their values, we calculated the total zIgG4/-zIgE ratio in comparison with total zIgG1/-zIgE ratio. (a) Significantly higher values of total zIgG4/-zIgE ratio were found in metastatic compared to primary tumor patients (*p* = .0009433) and to healthy donors (*p* = .02474). (b) No significant differences were found when we evaluated the total zIgG1/-zIgE ratio. One-way ANOVA with Tukey post-hoc test was performed. n.s. *P* > .05, # *P* < .1, * *P* < .05, *** *P* < .001, **** *P* < .0001

Recorded events by FACS Canto II flow cytometer (Becton Dickinson, Franklin Lakes, NJ, USA) were analyzed with the FlowJo^TM^ Software version 10.3 (FlowJo, LLC, Ashland, OR, USA). *FlowAI* plugin was used to identify anomalous events and clean the data by removing them. We designed a gate strategy (Repository Figure 2(a-d)) to identify tumor cells killed by ADCP (CD89-APC and CFSE-FITC double positive cells) and tumor cells killed by ADCC (Zombie Violet™-Pacific Blue and CFSE-FITC double positive cells).

### Statistical analysis

The graphs and the statistical analyses were performed with GraphPad Prism version 8.4.0 for Macintosh (GraphPad Software, La Jolla, CA, USA) and with R software (R version 3.6.2.). Validation of data from surface marker expression and cytokines/chemokines concentrations was done by repeated‐measures one‐way ANOVA and Tukey multiple comparison post‐hoc test. The level of statistical significance is defined as n.s. (not significant) *P* > .05, # *P* < .1, * *P* < .05, ** *P* < .01, *** *P* < .001, **** *P* < .0001.

Shapiro-Wilk test was used to test normality on the natural logarithm of laboratory data before applying Pearson’s correlation coefficient test with zIgG4/-zIgE and zIgG1/-zIgE.

The Youden index and the AUC were calculated using receiver operating characteristic (ROC) analysis to determine an optimal cutoff value for the zIgG4/-zIgE ratio to discriminate between metastatic and non-metastatic patients using a binormal method. We classified the donors regarding the presence of metastases including in the non-metastatic donors also the healthy donors: non-metastatic donors (class = 0), donors with metastases (class = 1). Then, we use a R script and the R package “ROCit” to derive all the parameters. A script in R was written to obtain all the statistical values and the graphs. The level of statistical significance was evaluated using the z-test: null hypotheses H_0_: AUC = AUC_0_; AUC_0_ = AUC with chance level (AUC = 0.5) .^32^ One-way ANOVA was applied for comparison of the zIgG4/-zIgE ratio between groups. All statistical results with a *p*-value <0.05 were considered significant.

We aimed at 80% power (alpha = .05) considering a one-way between-subjects ANOVA across three groups. Our calculations showed that the suggested number of participants was N = 60 and the study would be sensitive to effects of partial eta-squared (η^2^_p_) = 0.14, equating to a large effect. After exclusion of participants from calculations due to the reasons stated above (see “Study Population”), we had 45 participants across the three groups. In this case, the study is sensitive to effects of partial eta-squared (η^2^_p_) = 0.19; thus, the number of patients analyzed in the CRC group are able to describe statistically significant large effects .^33^

## Results

### Serum levels of IgG1, IgG4, and IgE immunoglobulins

**Figure 3.**
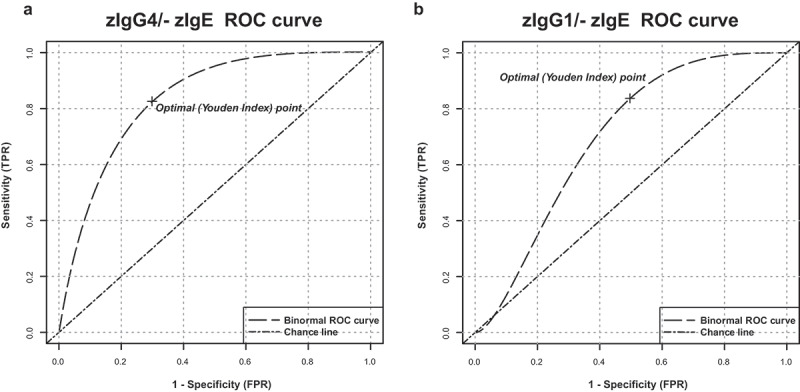
**Receiver operating characteristic (ROC) analysis of serum total zIgG4/-zIgE ratio and of total zIgG1/-zIgE ratio**. Receiver operating characteristic (ROC) analysis of total zIgG4/-zIgE and total zIgG1/-zIgE was performed using R package “ROCit”. (a) Binormal ROC curves were constructed for diagnostic performance considering total zIgG4/-zIgE in metastatic patients versus primary tumor patients. Youden Index point was calculated to determine the optimal cutoff for the diagnosis of metastases. Area under the ROC curves (AUC) = 0.83438, False Positive Rate (FPR) = 0.30, True Positive Rate (TPR) = 0.83. (b) Binormal ROC curves were constructed and Youden Index point was calculated also for total zIgG1/-zIgE in metastatic patients versus primary tumor patients. Area under the ROC curves (AUC) = 0.69702, False Positive Rate (FPR) = 0.50, True Positive Rate (TPR) = 0.83

After z-normalization, we measured significantly higher total IgG4 serum levels in patients with CRC diagnosed with metastatic compared to primary disease (*p* = .01109) (Figure 1). On the contrary, no significant changes were found after z-normalization of total IgG1 and IgE serum levels (Repository Figure 3(a-b)). The z-normalized total IgG4/IgE serum level ratio for each donor (zIgG4/-zIgE) was significantly higher in metastatic patients compared to healthy donors (*p* = .02474), and also significantly higher compared to patients with primary disease (*p* = .0009433) (Figure 2a). Comparison of zIgG1/-zIgE values showed no significant difference between groups (Figure 2b).

### Receiver operating characteristic (ROC) analysis of serum zIgG4/-zIgE and zIgG1/-zIgE

Receiver operating characteristics (ROC) analysis was performed to determine the cutoff value of total serum zIgG4/-zIgE levels that can be used to predict the metastases in diagnosed tumor patients (Figure 3(a-b) and Table 1). ROC analysis determined that the best cutoff value for the total serum zIgG4/-zIgE level to predict the presence of metastases was 1.04345 (*p* = 5.19081e-06). We also determined that, the area under the ROC curves (AUC) was 0.83438, the False Positive Rate (FPR) was 30% and the True Positive Rate (TPR) was 83%. On the contrary, the best cutoff value for the total serum zIgG1/-zIgE level, used as control, to predict the presence of metastases was 2.92326 (*p* = .04461). The area under the ROC curves (AUC) was 0.69702, the FPR was 50% and the TPR was 83%.

**Table 1.** ROC binormal analysis of CRC tumor patients

	AUC	CI	Cutoff	P value
**zIgG1/-zIgE**	0.69702	0.50474–0.8893	2.92326	0.04461
**zIgG4/-zIgE**	0.83438	0.69056–0.9782	1.04345	5.19081e-06

### Evaluation of IgG4 expression in tumor tissue sites

**Figure 4.**
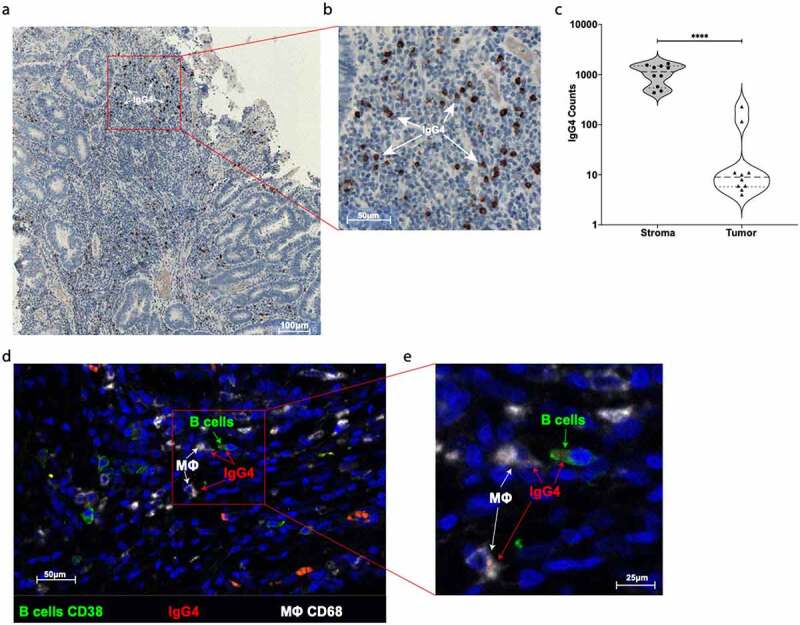
**Evaluation of IgG4 expression in CRC tumor site**. (a) One representative immunohistochemistry staining of formalin fixed paraffin embedded colon cancer slides against IgG4 (brown spots). (b) Highlighted area in red was magnified and IgG4 (brown spots) are marked with white arrows. (c) Quantification of IgG4 cell positivity in ten different tumor slides. We found significantly more IgG4 positive cells in the stroma site than in the tumor site microenvironment (Paired two tails Student’s t test, *p* < .0001). (d) One representative IF staining of formalin fixed paraffin embedded colon cancer slides against CD38 positive B cells (green), against IgG4 (red), and against CD68 positive macrophages cells (white). (e) Highlighted area in red was magnified to show the close contact between CD38^+^ green B cell expressing IgG4 (red spots) and CD68^+^ (white) macrophages

Immunohistochemical staining of CRC cancer tissues were evaluated by the TissueFAXS methodology. We found that IgG4 was mainly expressed in tumor stroma amongst all investigated tumor samples (Figure 4(a-c)). IF staining of tissue sections from CRC patients showed that IgG4, likely produced by the CD38 positive B cells, was found near CD68-positive monocytes/macrophages in tumors (Figure 4(d-e)).

Macrophages stimulated with IgG4 displayed M2b-like phenotype surface markers and cytokine production characteristics

Using a multicolor flow cytometric panel of monoclonal antibodies against the monocyte/macrophage lineage markers CD14, CD86, CD11b, CD163 and CD206, we investigated the phenotypic characteristics of human macrophages from healthy volunteers and from patients with CRC. We compared macrophages stimulated *ex vivo* with antibodies of different subclasses. Upon treatment of M2a cells with mIgG4, we applied dimensionality reduction to represent the shape of all events in two dimensions of the cell surface markers mentioned above. The obtained patterns differed from treatments between mIgG1 and mIgG1 stimulation (Repository Figure 6) in all groups, healthy donors, primary tumor patients and in metastatic patients. When considering the z-normalized geometric mean of intensity (zMFI) of each surface marker analyzed, we found that treatment of M2a with mIgG4 antibodies polarized macrophages toward an M2b‐like phenotype (Repository Figure 7). Both mIgG4 or mIgG1 induced a significant reduction of CD14, CD163 and CD206 expression in M2a cells from healthy, primary tumor, and metastatic patients compared to unstimulated M2a controls. This reduction in cell surface marker expression was more pronounced with mIgG4.

**Figure 5.**
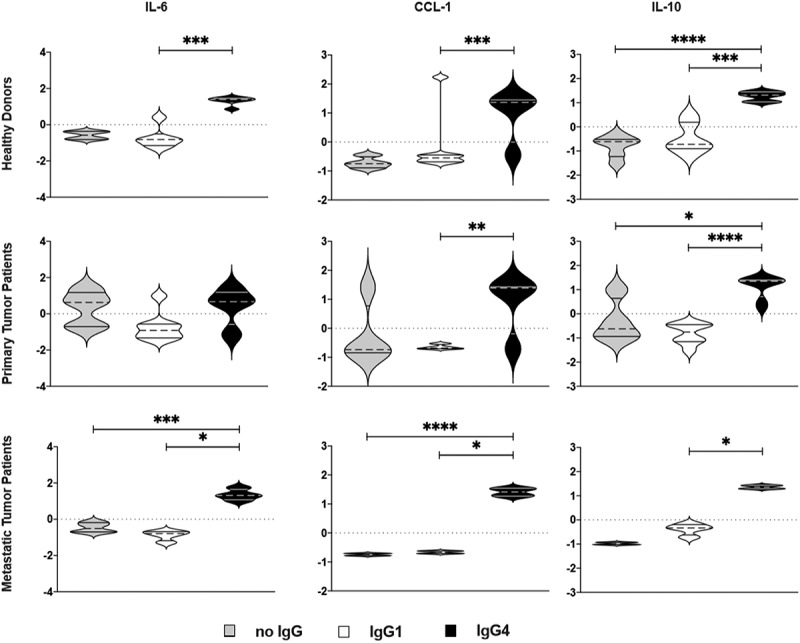
**Production of M2b-like cytokines/chemokines by M2a macrophages treated with mIgG1 or mIgG4 antibodies**. The supernatants of monocyte-derived macrophages (MDM) from the PBMCs of healthy donors or primary tumor patients or metastatic tumor patients were polarized *ex vivo* for 72 h. M2a macrophages treated ex vivo with plate-bound myeloma IgG1 (mIgG1) or plate-bound myeloma IgG1 (mIgG4) or left untreated were evaluated by cytokine ELISA for the detection of CCL-1, IL-10 and IL-6 production. Our results showed an increase of CCL-1, IL-10 and IL-6 production by M2a macrophages treated with mIgG4, similarly to M2b-like cytokine and chemokine production. These findings were true for all PBMC donor groups. The z‐normalization of pg/ml concentration was performed for each marker and each donor before the statistical validation. Gray bars: unstimulated M2a cells; white bars: M2a cells + mIgG1; black bars: M2a cells + mIgG4. The results from thirty-one independent experiments were combined for statistical analysis. One-way ANOVA with Tukey post-hoc test was performed. n.s. *P* > .05, * *P* < .05, ** *P* < .01, *** *P* < .001, **** *P* < .0001

In agreement, also the cytokine and chemokine expression by M2a macrophages when stimulated with mIgG4 containing immune complexes (ICs) (Figure 5) resembled a pattern typical of an M2b regulatory macrophage phenotype and was different from that in mIgG1 or no‐ICs stimulation samples for each tested cytokine.

U937 cell line: tumor cell line interaction when stimulated with immunotherapeutic IgG1 or IgG4 subclass

**Figure 6.**
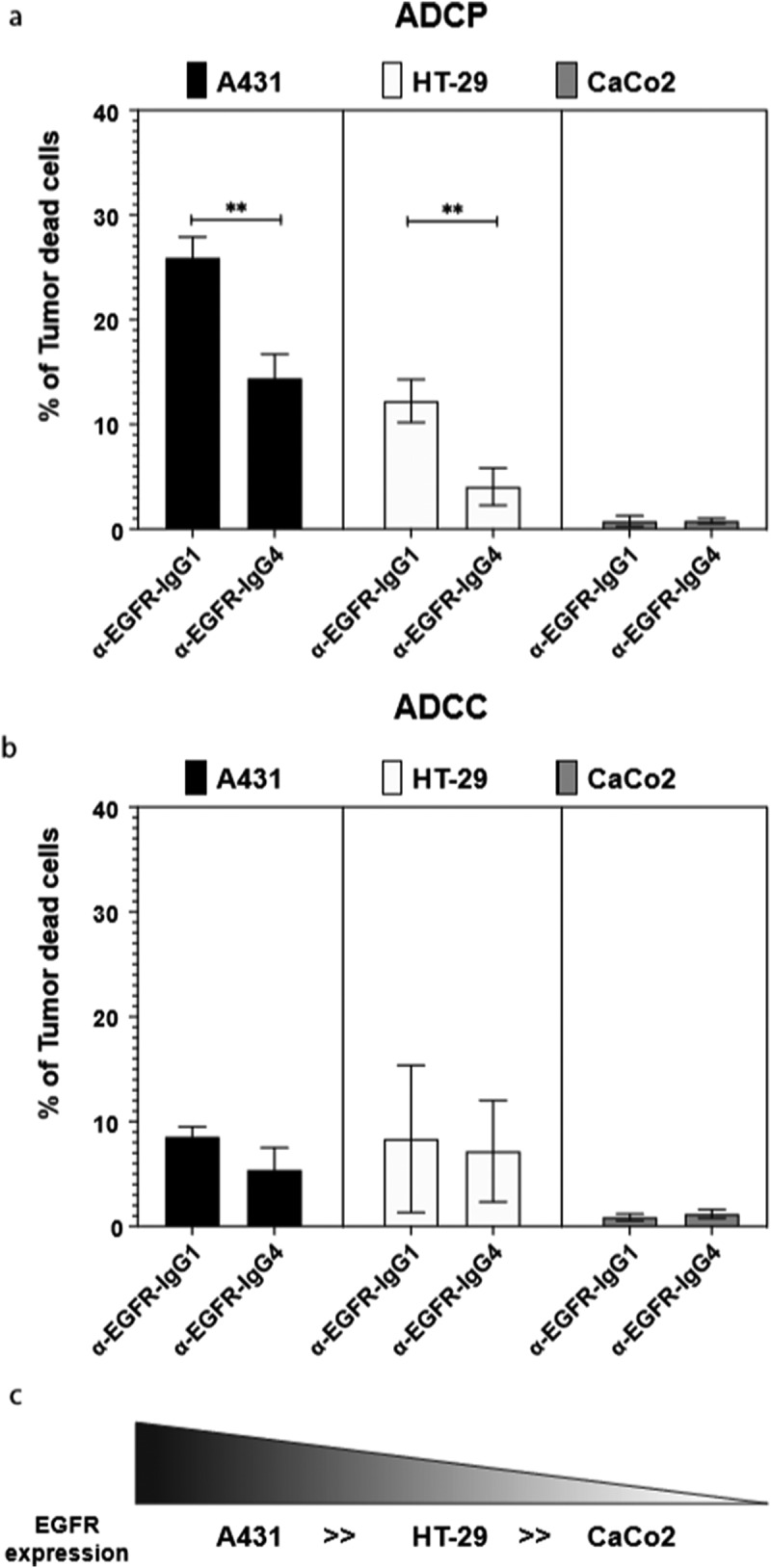
**Anti-human EGFR-IgG1 or IgG4 subclass effects on the ADCP and ADCC of tumor cell lines by human U937 monocytes**. U937 *in* vitro mediated mainly ADCP (a) and lower levels of ADCC (b) of EGFR differently expressing A431, HT-29 and CaCo2 tumor cell lines (c). The percentage of tumor dead cells was evaluated by flow cytometric analysis following the gating strategy represented on Repository figure 2. Black bars: A431 cells; white bars: HT-29 cells; gray bars: CaCo2 cells. The results from three independent experiments were combined for statistical analysis. One-way ANOVA with Tukey post-hoc test was performed. ** *P* < .01

We next used antibodies applied in clinical oncology for the *in vitro* stimulation of human U937 monocytic cells. Dependent on the IgG1 or IgG4 subclass, and directly correlating with the EGFR expression levels in the tested cell lines (A431 ≫ HT29 > CaCo2), the α-EGFR therapeutic anti-tumor antibody cetuximab displayed different capacity to trigger phagocytosis of tumor cells by human monocytes (Repository Figure 5). With IgG4, significantly lower percentages of tumor cells were killed by ADCP, compared to samples incubated with IgG1 (Figure 6).

## Discussion

IgG4 expression has been linked to immune tolerance in allergic diseases^34^ and shown to increase upon prolonged and/or high-dose antigenic stimulation in atopic patients undergoing allergen immunotherapy. Emerging findings also indicate that IgG4 is highly expressed in certain tumor tissue types^19,22^ and its presence correlates with poor prognosis in some malignancies. In this study we report significantly higher serum IgG4 levels and higher zIgG4/-zIgE ratios in metastatic compared to non-metastatic CRC patients. This shift in proportions between serum IgG4 and IgE levels in metastatic patients indicates both an increase of circulating IgG4 and a decrease in circulating IgE levels, which is most strikingly illustrated as an elevated zIgG4/-zIgE ratio. Furthermore, we recently summarized the current knowledge on macrophage re-polarization and cytokine production in the context of potential pro-tumorigenic functions of IgG4, ^35^ by the reshaping of the tumor microenvironment. Here, in colon tissues from CRC patients, we report that IgG4 was expressed in close proximity to macrophages. We present data in support of a critical role for IgG4 in driving human macrophages, such as those found infiltrating tumors, toward featuring cell surface molecule characteristics and secretory capacity of an alternatively activated, immune-suppressive state.

Several pro- and anti-inflammatory mediators, which can be synthesized and released by macrophages, are involved in the process of tumorigenesis and metastasis. Pro-inflammatory cytokines (e.g. TNF-α, IL-6), particularly in chronic inflammation, can lead to reactive oxygen species-derived DNA-damage, resulting in tumor promotion. Anti-inflammatory cytokines (e.g. IL-10, TGF-β) support tumor expansion via immune evasion, with the invasive properties being promoted by both anti-inflammatory, that is epithelial-mesenchymal transition being enabled by TGF-ß, and pro-inflammatory cytokines, which are also involved in angiogenesis and metastasis. IL-8 and other chemokines are involved in tumor cell migration. Serum IL-6 or IL-10 are discussed as potential markers of tumorigenesis stages and tumor-associated prognosis .^36^ While progress has been made in the understanding of the mechanisms of these cytokines in the tumorigenic process, establishing a relationship between cytokine expression and disease progression, survival, and response to therapy remains a major challenge.

Cancer cells secrete chemoattractants (e.g., M-CSF, PDGF) which recruit monocytes from the periphery to the tumor site. Monocytes differentiate into TAMs with capacity to secrete pro- and also anti-inflammatory mediators depending on their differentiation states .^3^ While initially M1 macrophages can inhibit tumor growth, later M2-like macrophages create a favorable pro-tumorigenic microenvironment and are a source of high levels of immunosuppressive cytokines such as IL-10 .^2,24,25^ Importantly, IL-10 plays a role in enhancing IgG4 production on site. In healthy volunteer, and patient groups with primary or metastatic CRC, IgG4-stimulated M2 macrophages manifested lower surface CD206, CD163, and CD14 expression and higher CCL-1, IL-10, and IL-6 production, in an isotype-specific manner (Figure 6), a profile strongly resembling the M2b‐phenotype [17] (Repository Figure 7), and consistent with that we previously reported in an allergy model .^23^

The release of immune-modulating IgG4 antibodies from the tumor stroma may promote a tolerogenic microenvironment by disruption of TAM mediated anti-tumor mechanisms, such as ADCC and ADCP. To assess whether exogenous IgG4 antibodies could similarly modify the effector response of macrophages, we used two isotype variants of the anti-EGFR therapeutic anti-tumor antibody cetuximab, both with the same variable regions, but with γ1 or γ4 constant domains. IgG4 (Figure 6) was less potent in eradicating EGFR-expressing tumor cells by ADCP than IgG1. Thus, TAMs may not only be modulated by endogenous IgG4 toward the immunosuppressive M2b-like phenotype, but are also less effective in becoming activated to kill tumor cells by exogenous IgG4 antibodies applied in clinical oncology .^37^ The potential effector functions of therapeutic antibodies may be a consideration in the design of therapeutic agents including potentially checkpoint inhibitor agents such as anti-PD1 IgG4 antibodies Nivolumab or Pembrozilumab .^38,39^

In contrast to IgG4, IgE cross-linking on densely displayed, overexpressed tumor antigens forming TAMPs^40^ may trigger effector cell activation and release of mast cell-derived mediators, such as TNF-α. This can drive innate effector cell responses which can contribute to anti-tumor inflammation. The promising research on the role of IgE in cancer has led to the establishment of the emerging field of AllergoOncology, investigating multifaceted functions of IgE in cancer [27, 28]. Interestingly, IgE may reprogram monocytes and macrophages toward cytotoxic functions, ^7,41^ and the first therapeutic application of tumor-specific IgE antibody MOv18 in ovarian cancer patients is presently ongoing in a registered clinical trial (NCT02546921).

Overall, we provide evidence for a detrimental role of IgG4 in CRC from combined in vitro and patient-derived phenotypic and functional evaluations. We report here that: i) elevated IgG4-expressing cells occur especially in close vicinity to macrophages in CRC tissue; ii) higher levels of IgG4/-IgE ratios were found in metastatic compared to primary CRC disease, iii) in macrophages derived from healthy volunteers and different CRC patient groups, IgG4 stimulation could induce an immunoregulatory M2b-like macrophage phenotype, and iv) tumor antigen-specific IgG4 showed impaired antibody-mediated phagocytosis of tumor cells compared with IgG1. Taken together our findings support a role for IgG4 in impairing macrophage activation which may have clinical relevance in CRC. The fact that macrophages in CRC patients have the same polarization plasticity as those macrophages from healthy donors, gives hope that it may be possible to immunologically manipulate and potentially diminish the tumor-promoting functions of IgG4 in the future.

### Limitations

At this point, we cannot clearly extrapolate our findings to other tumor entities. While in preliminary studies, we found abundance of IgG4+ infiltrating cells in bladder, kidney and prostate cancers, our retrospective analyses of available samples from these cancer cohorts allowed for identification of similar trends (Supplementary Material 1). These studies must therefore be repeated in larger patient cohorts with a sufficient sample size.

### Conclusions

IgG4 is highly expressed in CRC and drives M2a macrophages to a tolerogenic M2b-like phenotype. Our data indicate that the presence of IgG4 in CRC can shape an immunosuppressive microenvironment, resulting in cancer progression and poor prognostic outcomes. Total serum zIgG4/-zIgE ratio might prove a useful indicator of a metastatic CRC stage.

## Supplementary Material

Supplemental Material

## Figures and Tables

**Table:** 

ADCC	antibody-dependent cell-mediated cytotoxicity
ADCP	antibody-dependent cell-mediated phagocytosis
Breg/Bregs	B regulatory cell/s
CCL1	CC‐chemokine ligand 1
CCR8	C‐chemokine receptor 8
CRC	colorectal cancer
FcγR	Fc gamma receptor
Ig	immunoglobulin
ICs	immune complexes
IL	interleukin
M-CSF	macrophage colony-stimulating factor
MDMs	monocyte‐derived macrophages
MFI	geometric mean of fluorescence intensity
MMP	matrix metalloprotease
NK	natural killer cell
PDGF	platelet-derived growth factor
rh‐	recombinant human
TAMs	tumor-associated macrophages
TAMPs	tumor-associated molecular patterns
TATE	tumor-associated tissue eosinophilia
Th	T-helper cell
TNFα	tumor necrosis factor alpha
TGFβ	tumor growth factor beta
Treg/Tregs	T regulatory cell/s

## References

[1] Komi DEA, Redegeld FA. Role of mast cells in shaping the tumor microenvironment. Clin Rev Allergy Immunol. 2019;58(3):313-25. 10.1007/s12016-019-08753-w PMC7244463 31256327

[2] Chanmee T, Ontong P, Konno K, Itano N. Tumor-associated macrophages as major players in the tumor microenvironment. Cancers (Basel). 2014;6(3):1670–12. doi:10.3390/cancers6031670. 25125485 PMC4190561

[3] Lewis CE, Pollard JW. Distinct role of macrophages in different tumor microenvironments. Cancer Res. 2006;66(2):605–612. doi:10.1158/0008-5472.CAN-05-4005. 16423985

[4] Vitale I, Manic G, Coussens LM, Kroemer G, Macrophages GL. Metabolism in the tumor microenvironment. Cell Metab. 2019;30(1):36–50. doi:10.1016/j.cmet.2019.06.001. 31269428

[5] Saraiva AL, Carneiro F. New insights into the role of tissue eosinophils in the progression of colorectal cancer: a literature review. Acta Med Port. 2018;31(6):329–337. doi:10.20344/amp.10112. 30020878

[6] Simon SCS, Utikal J, Umansky V. Opposing roles of eosinophils in cancer. Cancer Immunol Immunother. 2019;68(5):823–833. doi:10.1007/s00262-018-2255-4. 30302498 PMC11028063

[7] Pellizzari G, Hoskin C, Crescioli S, Mele S, Gotovina J, Chiaruttini G, Bianchini R, Ilieva K, Bax HJ, Papa S, et al. IgE re-programs alternatively-activated human macrophages towards pro-inflammatory anti-tumoural states. EBioMedicine. 2019;43:67–81. doi:10.1016/j.ebiom.2019.03.080. 30956175 PMC6562024

[8] Lin AA, Freeman AF, Nutman TB. IL-10 indirectly downregulates IL-4-induced IgE production by human B cells. Immunohorizons. 2018;2(11):398–406. doi:10.4049/immunohorizons.1800076. 31026808 PMC8890443

[9] Jeannin P, Lecoanet S, Delneste Y, Gauchat JF, Bonnefoy JY. IgE versus IgG4 production can be differentially regulated by IL-10. J Immunol. 1998;160:3555–3561. 9531318

[10] Nirula A, Glaser SM, Kalled SL, Taylor FR. What is IgG4? A review of the biology of a unique immunoglobulin subtype. Curr Opin Rheumatol. 2011;23(1):119–124. doi:10.1097/BOR.0b013e3283412fd4. 21124094

[11] Karagiannis P, Gilbert AE, Josephs DH, Ali N, Dodev T, Saul L, Correa I, Roberts L, Beddowes E, Koers A, et al. IgG4 subclass antibodies impair antitumor immunity in melanoma. J Clin Invest. 2013;123(4):1457–1474. doi:10.1172/JCI65579. 23454746 PMC3613918

[12] Crescioli S, Correa I, Karagiannis P, Davies AM, Sutton BJ, Nestle FO, Karagiannis SN. IgG4 characteristics and functions in cancer immunity. Curr Allergy Asthma Rep. 2016;16(1):7. doi:10.1007/s11882-015-0580-7. 26742760 PMC4705142

[13] Peppas I, George G, Sollie S, Josephs DH, Hammar N, Walldius G, Karagiannis SN, Van Hemelrijck M. Association of serum immunoglobulin levels with solid cancer: a systematic review and meta-analysis. Cancer Epidemiol Biomarkers Prev. 2020;29(3):527–538. doi:10.1158/1055-9965.EPI-19-0953. 31915145

[14] Su Y, Sun W, Wang C, Wu X, Miao Y, Xiong H, Bai L, Dong L. Detection of serum IgG4 levels in patients with IgG4-related disease and other disorders. PLoS One. 2015;10(4):e0124233. doi:10.1371/journal.pone.0124233. 25885536 PMC4401680

[15] Yamamoto M, Takahashi H, Tabeya T, Suzuki C, Naishiro Y, Ishigami K, Yajima H, Shimizu Y, Obara M, Yamamoto H, et al. Risk of malignancies in IgG4-related disease. Mod Rheumatol. 2012;22(3):414–418. doi:10.3109/s10165-011-0520-x. 21894525

[16] Hirano K, Tada M, Sasahira N, Isayama H, Mizuno S, Takagi K, Watanabe T, Saito T, Kawahata S, Uchino R, et al. Incidence of malignancies in patients with IgG4-related disease. Intern Med. 2014;53(3):171–176. doi:10.2169/internalmedicine.53.1342. 24492683

[17] Shimo T, Yao M, Takebe Y, Ono Y, Obata K, Kurio N, Ibaragi S, Yoshioka N, Kishimoto K, Yanagi Y, et al. A case of adenoid cystic carcinoma associated with IgG4-related disease. Int J Surg Case Rep. 2015;10:12–16. doi:10.1016/j.ijscr.2015.01.022. 25781921 PMC4429946

[18] Deshpande V. IgG4-related disease of the gastrointestinal tract: a 21st century Chameleon. Arch Pathol Lab Med. 2015;139(6):742–749. doi:10.5858/arpa.2014-0181-RA. 26030243

[19] Kamisawa T, Chen PY, Tu Y, Nakajima H, Egawa N, Tsuruta K, Okamoto A, Hishima T. Pancreatic cancer with a high serum IgG4 concentration. World J Gastroenterol. 2006;12(38):6225–6228. doi:10.3748/wjg.v12.i38.6225. 17036401 PMC4088123

[20] Caturegli P, Kuppers RC, Mariotti S, Burek CL, Pinchera A, Ladenson PW, Rose NR. IgG subclass distribution of thyroglobulin antibodies in patients with thyroid disease. Clin Exp Immunol. 1994;98(3):464–469. doi:10.1111/j.1365-2249.1994.tb05514.x. 7994910 PMC1534515

[21] Lucas SD, Karlsson-Parra A, Nilsson B, Grimelius L, Akerstrom G, Rastad J, Juhlin C.. Tumor-specific deposition of immunoglobulin G and complement in papillary thyroid carcinoma. Hum Pathol. 1996;27(12):1329–1335. doi:10.1016/S0046-8177(96)90346-9. 8958307

[22] Miyatani K, Saito H, Murakami Y, Watanabe J, Kuroda H, Matsunaga T, Fukumoto Y, Osaki T, Nakayama Y, Umekita Y, et al. A high number of IgG4-positive cells in gastric cancer tissue is associated with tumor progression and poor prognosis. Virchows Arch. 2016;468(5):549–557. doi:10.1007/s00428-016-1914-0. 26951261

[23] Bianchini R, Roth-Walter F, Ohradanova-Repic A, Flicker S, Hufnagl K, Fischer MB, Stockinger H, Jensen-Jarolim E. IgG4 drives M2a macrophages to a regulatory M2b-like phenotype: potential implication in immune tolerance. Allergy. 2019;74(3):483–494. doi:10.1111/all.13635. 30338531 PMC6492166

[24] Ruffell B, Coussens LM. Macrophages and therapeutic resistance in cancer. Cancer Cell. 2015;27(4):462–472. doi:10.1016/j.ccell.2015.02.015. 25858805 PMC4400235

[25] Pollard JW. Tumour-educated macrophages promote tumour progression and metastasis. Nat Rev Cancer. 2004;4(1):71–78. doi:10.1038/nrc1256. 14708027

[26] Martinez FO, The GS. M1 and M2 paradigm of macrophage activation: time for reassessment. F1000Prime Rep. 2014;6:13. doi:10.12703/P6-13. 24669294 PMC3944738

[27] Mallmann S, S. V, Schultze JL. Macrophages in human cancer. Current and future aspects. Atlas Genet Cytogenet Oncol Haematol. 2012. doi:10.4267/2042/48157.

[28] Murray PJ, Allen JE, Biswas SK, Fisher EA, Gilroy DW, Goerdt S, Gordon S, Hamilton JA, Ivashkiv LB, Lawrence T, et al. Macrophage activation and polarization: nomenclature and experimental guidelines. Immunity. 2014;41(1):14–20. doi:10.1016/j.immuni.2014.06.008. 25035950 PMC4123412

[29] Mechtcheriakova D, Sobanov Y, Holtappels G, Bajna E, Svoboda M, Jaritz M, Bachert C, Jensen-Jarolim E. Activation-induced cytidine deaminase (AID)-associated multigene signature to assess impact of AID in etiology of diseases with inflammatory component. PLoS One. 2011;6(10):e25611. doi:10.1371/journal.pone.0025611. 21984922 PMC3184987

[30] Singer J, Weichselbaumer M, Stockner T, Mechtcheriakova D, Sobanov Y, Bajna E, Wrba F, Horvat R, Thalhammer JG, Willmann M, et al. Comparative oncology: erbB-1 and ErbB-2 homologues in canine cancer are susceptible to cetuximab and trastuzumab targeting. Mol Immunol. 2012;50(4):200–209. doi:10.1016/j.molimm.2012.01.002. 22424313 PMC3318186

[31] Bracher M, Gould HJ, Sutton BJ, Dombrowicz D, Karagiannis SN. Three-colour flow cytometric method to measure antibody-dependent tumour cell killing by cytotoxicity and phagocytosis. J Immunol Methods. 2007;323(2):160–171. doi:10.1016/j.jim.2007.04.009. 17531261

[32] Hajian-Tilaki K. Receiver operatingCharacteristic (ROC) curve analysis for medical diagnostic test evaluation. Caspian J Intern Med. 2013;4:627–635. 24009950 PMC3755824

[33] Lenhard W, Lenhard A. Calculation of effect sizes. Dettelbach: Germany; 2016. Retrieved from: https://www.psychometrica.de/effect_size.html.

[34] Mitsias DI, Xepapadaki P, Makris M, Papadopoulos NG. Immunotherapy in allergic diseases - improved understanding and innovation for enhanced effectiveness. Curr Opin Immunol. 2020;66:1–8. doi:10.1016/j.coi.2020.02.005. 32272340

[35] Bianchini R, Karagiannis SN, Jordakieva G, Jensen-Jarolim E. The role of IgG4 in the fine tuning of tolerance in IgE-mediated allergy and cancer. Int J Mol Sci. 2020;21:14. doi:10.3390/ijms21145017. PMC7404042 32708690

[36] Landskron G, De la Fuente M, Thuwajit P, Thuwajit C, Hermoso MA. Chronic inflammation and cytokines in the tumor microenvironment. J Immunol Res. 2014;2014:149185. doi:10.1155/2014/149185. 24901008 PMC4036716

[37] Kretschmer A, Schwanbeck R, Valerius T, Rosner T. Antibody isotypes for tumor immunotherapy. Transfus Med Hemother. 2017;44:320–326. 29070977 10.1159/000479240 PMC5649311

[38] Dahan R, Sega E, Engelhardt J, Selby M, Korman AJ, Ravetch JV. FcgammaRs modulate the anti-tumor activity of antibodies targeting the PD-1/PD-L1 Axis. Cancer Cell. 2015;28(4):543. doi:10.1016/j.ccell.2015.09.011. 28854351

[39] Chen X, Song X, Li K, Zhang T. FcgammaR-binding is an important functional attribute for immune checkpoint antibodies in cancer immunotherapy. Front Immunol. 2019;10:292. doi:10.3389/fimmu.2019.00292. 30863404 PMC6399403

[40] Jensen-Jarolim E, Mechtcheriakova D, Pali-Schoell I. The targets of IgE: allergen-associated and tumor-associated molecular patterns. Penichet ML, Jensen-Jarolim E. editors, Cancer and IgE: introducing the concept of allergoOncology. Totowa (NJ): Humana Press; 2010; 231–254.

[41] Nakamura M, Souri EA, Osborn G, Laddach R, Chauhan J, Stavraka C, Lombardi S, Black A, Khiabany A, Khair DO, et al. IgE activates monocytes from cancer patients to acquire a pro-inflammatory phenotype. Cancers (Basel). 2020;12:11. doi:10.3390/cancers12113376. PMC7698027 33203088

