# Supplementary material for: IgG4 induces tolerogenic M2-like macrophages and correlates with disease progression in colon cancer
Source: Oncoimmunology. 2021 Feb 8;10(1):1880687. doi: 10.1080/2162402X.2021.1880687 (PMC7889146; doi:10.1080/2162402X.2021.1880687)
Supplement: Supplemental Material [file KONI_A_1880687_SM8285.docx]

*Supplementary Material*

*Methods*

*Complementary analyses in other cancer cohorts: bladder, kidney and prostate cancer*Patients were recruited between 2018 and 2019 at the in-patient ward of the Urology Clinic of the S.M.M. Hospital, University of Perugia, Italy, if they fulfilled the inclusion criteria described below. The study was approved by the local Ethics Committee Board “CEAS”, Umbria, Italy (Nr. 3193/18, clear cell renal cell carcinoma patients; 3171/18 bladder cancer patients; Nr. 3154/18, prostate carcinoma patients) and volunteering participants were only included after gaining written informed consent. All samples were evaluated anonymously. Participants were in hospital for surgery in recently diagnosed clear cell renal cell carcinoma (ccRCC), bladder cancer (BC) or prostate cancer (PC) with (=TxNxM1) and without (=TxNxM0) systemic metastasis at initial diagnosis. Clinical characteristics, laboratory parameters, sociodemographic and follow-up data were obtained from clinical records. The blood samples were obtained before surgery, prepared and frozen at -20°C and rapidly sent to a research lab for analysis by a blinded study member (GN).

## *Inclusion criteria:*

• age between 18-85 years

• recent initial cancer diagnosis, without any preceding or neo-adjuvant therapy

• adequate Italian language skills

• signed declaration of consent

## *Exclusion criteria:*

• innate or acquired immune deficiencies

• ongoing potentially immune-modulating therapies

• any other kind of malignancy in patient history

• previously diagnosed IgG4-associated disease and/or known serum IgG4 elevation.

*Results*

## *Study population: complementary cancer cohort*

Forty-three patients (n=44) were recruited between 2018 and 2019 at the in-patient ward of the Urology Clinic of the S.M.M. Hospital, University of Perugia, Italy. Blood samples were obtained after gaining written informed consent and rapidly analyzed in a blinded manner. All the forty-three patients recruited were eligible for analyses.

Participants were in hospital for surgery and were diagnosed as:

- Twenty patients (n=21) with bladder cancer (BC) (81% male; age 71.9±9 years);
  - without systemic metastasis at initial diagnosis (=TxNxM0) (n=13);
  - with systemic metastasis at initial diagnosis (=TxNxM1) (n=8);
- Ten (n=10) with clear cell renal cell carcinoma (ccRCC) (60% male; age 60.7±13.9 years);
  - without systemic metastasis at initial diagnosis (=TxNxM0) (n=4);
  - with systemic metastasis at initial diagnosis (=TxNxM1) (n=6);
- Thirteen (n=13) with prostate cancer (PC) (100% male; age 70.2±5.2 years);
  - without systemic metastasis at initial diagnosis (=TxNxM0) (n=8);
  - with systemic metastasis at initial diagnosis (=TxNxM1) (n=5);

## *Sera levels of IgG1, IgG4 and IgE immunoglobulins*

The analyses of zIgG4/zIgE and zIgG1/zIgE conducted in these complementary cancer cohorts showed a similar trend of lower serum zIgG4/zIgE values, particularly in metastatic kidney cancer compared to non-metastatic kidney tumor (p=0.0956)(Repository Figure 4 a-c and Table E1-E3).

**Repository Figures 4. Evaluation of total zIgG4/zIgE ratio**. Total IgG4, IgG1 and IgE levels of healthy donors, primary cancer patients and metastatic patients were measured. After z-normalization of all their values, we calculated the total zIgG4/zIgE ratio in comparison with total zIgG1/zIgE ratio in (a) bladder cancer, (b) kidney cancer and (c) prostate cancer. One-way ANOVA with Tukey post-hoc test was performed. n.s. P > 0.05, # P < 0.1, * P < 0.05, ** P < 0.01, *** P < 0.001, **** P < 0.0001.

## *Receiver operating characteristic (ROC) analysis of serum zIgG4/zIgE and zIgG1/zIgE*

The analyses on the total serum zIgG4/zIgE levels and the total serum zIgG1/zIgE levels in these complementary cancer cohorts using total serum zIgG4/zIgE level cut-off for discriminating between metastatic and non-metastatic tumor patients (bladder cut-off=-1.45711, p= 0.08264; kidney cut-off=1.10455, p=0.07754) showed a trend in bladder and kidney cancer only. These results of ROC analyses are reported in the online repository data (Table E4-E6).

**Table E1. One-way ANOVA with Tukey post-hoc test of Bladder cancer.**

|  | **Healthy Donors**  **vs.**  **Primary tumor patients** | **Healthy Donors**  **vs.**  **Metastatic tumor patients** | **Primary tumor patients**  **vs.**  **Metastatic tumor patients** |
| --- | --- | --- | --- |
| **zIgG1** | 0.859 | 0.738 | 0.966 |
| **zIgG4** | 0.890 | 0.979 | 0.826 |
| **zIgE** | 0.334 | 0.378 | 1.00 |
| **zIgG1/zIgE** | 0.555 | 0.746 | 0.974 |
| **zIgG4/zIgE** | 0.198 | 0.945 | 0.168 |

**Table E2. One-way ANOVA with Tukey post-hoc test of Kidney cancer.**

|  | **Healthy Donors**  **vs.**  **Primary tumor patients** | **Healthy Donors**  **vs.**  **Metastatic tumor patients** | **Primary tumor patients**  **vs.**  **Metastatic tumor patients** |
| --- | --- | --- | --- |
| **zIgG1** | 0.641 | 0.789 | 0.419 |
| **zIgG4** | 0.299 | 0.599 | 0.123 |
| **zIgE** | 0.671 | 0.990 | 0.794 |
| **zIgG1/zIgE** | 0.440 | 0.996 | 0.494 |
| **zIgG4/zIgE** | 0.127 | 0.847 | 0.0956 |

**Table E3. One-way ANOVA with Tukey post-hoc test of Prostate cancer.**

|  | **Healthy Donors**  **vs.**  **Primary tumor patients** | **Healthy Donors**  **vs.**  **Metastatic tumor patients** | **Primary tumor patients**  **vs.**  **Metastatic tumor patients** |
| --- | --- | --- | --- |
| **zIgG1** | 0.490 | 0.984 | 0.559 |
| **zIgG4** | 0.981 | 0.838 | 0.790 |
| **zIgE** | 0.475 | 0.482 | 0.987 |
| **zIgG1/zIgE** | 0.675 | 0.798 | 0.997 |
| **zIgG4/zIgE** | 0.431 | 0.833 | 0.897 |

**Table E4. ROC binormal analysis of Bladder tumor patients**

|  | **AUC** | **CI** | **Cutoff** | **P value** |
| --- | --- | --- | --- | --- |
| **zIgG1/zIgE** | 0.56405 | 0.31267 - 0.81542 | -3.08651 | 0.61747 |
| **zIgG4/zIgE** | 0.70003 | 0.47412 - 0.9259 | -1.45711 | 0.08264 |

**Table E5. ROC binormal analysis of Kidney tumor patients**

|  | **AUC** | **CI** | **Cutoff** | **P value** |
| --- | --- | --- | --- | --- |
| **zIgG1/zIgE** | 0.72408 | 0.31267 - 0.81542 | -1.34174 | 0.20459 |
| **zIgG4/zIgE** | 0.78557 | 0.46847 - 0.99 | 1.10455 | 0.07754 |

**Table E6. ROC binormal analysis of Prostate tumor patients**

|  | **AUC** | **CI** | **Cutoff** | **P value** |
| --- | --- | --- | --- | --- |
| **zIgG1/zIgE** | 0.61479 | 0.29596 - 0.81542 | -3.90289 | 0.48037 |
| **zIgG4/zIgE** | 0.63137 | 0.31658 - 0.93362 | 4.06969 | 0.41336 |
